# *Borrelia burgdorferi* Sensu Stricto DNA in Field-Collected *Haemaphysalis longicornis* Ticks, Pennsylvania, United States

**DOI:** 10.3201/eid2702.201552

**Published:** 2021-02

**Authors:** Keith J. Price, Christine B. Graham, Bryn J. Witmier, Holly A. Chapman, Brooke L. Coder, Christian N. Boyer, Erik Foster, Sarah E. Maes, Ying Bai, Rebecca J. Eisen, Andrew D. Kyle

**Affiliations:** Pennsylvania Department of Environmental Protection, Harrisburg, Pennsylvania, USA (K.J. Price, B.J. Witmier, H.A. Chapman, B.L. Coder, C.N. Boyer, A.D. Kyle);; Centers for Disease Control and Prevention, Fort Collins, Colorado, USA (C.B. Graham, E. Foster, S.E. Maes, Y. Bai, R.J. Eisen)

**Keywords:** Asian longhorned tick, Borrelia burgdorferi, bacteria, Haemaphysalis longicornis, invasive species, Lyme disease, PCR, Pennsylvania, ticks, United States

## Abstract

We collected questing *Haemaphysalis longicornis* ticks from southeastern counties of Pennsylvania, USA. Of 263 ticks tested by PCR for pathogens, 1 adult female was positive for *Borrelia burgdorferi* sensu stricto, yielding a 0.4% infection rate. Continued monitoring of this invasive tick is essential to determine its public health role.

*Borrelia burgdorferi* sensu stricto is the causative agent of Lyme disease, the most commonly reported vectorborne disease in North America (*1*). In Pennsylvania, which is first in the United States in the number of reported Lyme disease cases, the spirochete has been identified in nearly 50% of adult *Ixodes scapularis* ticks, the primary vector (*2*). In 2018, Pennsylvania initiated a statewide active surveillance program to monitor tick distribution and density, by county, and tickborne pathogen prevalence. Although focused primarily on collecting and testing *Ixodes scapularis* ticks, initial surveillance efforts recovered, among other species, *Haemaphysalis longicornis* (Asian longhorned tick), an exotic species recently detected in North America (*3*), providing quantitative records of their presence in Pennsylvania public lands (*4*).

Since its US discovery in New Jersey during 2017, the number of states that have detected *H. longicornis* ticks has increased rapidly. In its native range, *H. longicornis* ticks have been found to carry a variety of pathogens endemic to Pennsylvania, including *B. burgdorferi* (*5*). However, because the ecologic characteristics and the pathogen diversity and prevalence of *H. longicornis* ticks in the United States are understudied, potential epidemiologic risks there remain unknown. We report surveillance program data on the presence of pathogen-infected *H. longicornis* in public areas in Pennsylvania.

## The Study

We performed surveillance activities weekly in 38 Pennsylvania counties during May 1–September 6, 2019, capturing peak nymphal *I. scapularis* ticks, in addition to adult and nymphal *H. longicornis* tick densities (*6*). Sampling sites, primarily high-use public areas in deciduous forests, were selected for high risk of recreational and occupational tick encounters and suitable *I. scapularis* and reported *H. longicornis* tick habitat (*6*).

Collection processes were standardized to minimize spatial and temporal bias. We collected questing ticks by dragging a 1 m^2^ white felt cloth over vegetation and leaf litter for 100–600 m. We examined cloths every 10 m and transferred recovered ticks into vials containing 80% ethanol, which we shipped to a central laboratory where they were stored at −80°C until being identified using morphological keys.

We tested the majority (84%) of collected *H. longicornis* nymphs and adults for pathogens, then retained the rest as voucher specimens. We prepared DNA extracts from individual *H. longicornis* tick homogenates on the KingFisher Flex Purification System with the MagMAX CORE Nucleic Acid Purification Kit (ThermoFisher Scientific, https://www.thermofisher.com). We tested each extract for *B. burgdorferi* sensu stricto, *B. mayonii*, *B. miyamotoi*, and *Babesia microti* using probe-based real-time PCR assays comprising multiple targets for each pathogen (Table). We amplified a segment of the *Borrelia* dipeptidyl aminopeptidase (PepX) gene using seminested PCR and sequenced it to confirm *B. burgdorferi* sensu stricto–positive specimens. We followed real-time PCR and PepX amplification protocols published elsewhere (*9*). We amplified and sequenced a 667-nt fragment of the cytochrome oxidase subunit I (COI) gene using primers LCO1490 and HCO2198 (*11*) to confirm the tick species of positive specimens. The PCR mixture (25 µL) contained forward and reverse primers at a final concentration of 0.4 µmol and 5 µL of DNA template. Thermocycling conditions followed protocols published elsewhere (*11*). COI and PepX amplicons were sequenced as described elsewhere (*9*).

**Table:** Pathogen targets included in real-time PCR testing of individual *Haemaphysalis longicornis* ticks, Pennsylvania, USA *†

PCR target	Pathogen				
	*Borrelia burgdorferi* sensu stricto	*B. mayonii*	*B. miyamotoi*	*Babesia microti*	Reference
*Borrelia* 16S rDNA	‡	‡	‡	NA	(*7*)
*B. burgdorferi* sensu lato *fliD*	‡	‡	NA	NA	(*8*)
*B. burgdorferi* sensu stricto *oppA2*	‡	NA	NA	NA	(*9*)
*B. mayonii oppA2*	NA	‡	NA	NA	(*9*)
*Borrelia miyamotoi purB*	NA	NA	‡	NA	(*9*)
*B. miyamotoi glpQ*	NA	NA	‡	NA	(*9*)
*B. microti sa1*	NA	NA	NA	‡	(*10*)
*B. microti* 18S rDNA	NA	NA	NA	‡	(*10*)

*fliD, flagellin gene; NA, not applicable; *oppA2*, oligopeptide permease periplasmic A2 gene; *purB*, adenylosuccinate lyase gene; *glpQ*, glycerophosphodiester phosphodiesterase gene; *sa1*, secreted antigen 1 gene. †A sample was considered positive for a pathogen only if it was positive for all associated targets. ‡Targets associated with each pathogen.

## Results

A total of 668 *H. longicornis* ticks (356 larvae, 166 nymphs, 146 adults) were collected from 4 counties in southeastern Pennsylvania (Figure). During the same period, 265 *I. scapularis* ticks (174 larvae, 78 nymphs, 13 adults) were collected from the same 4 counties. Of the subset of *H. longicornis* ticks tested by using real-time PCR (n = 263), 1 (0.4%) adult female collected from a county park in Bucks County on June 14, 2019 was positive for *B. burgdorferi* sensu stricto. A 570-nt segment of the PepX gene from this specimen was identical to *B. burgdorferi* sensu stricto reference sequences (GenBank accession nos. CP002312.1:657467–658036). The COI gene fragment from this tick showed 99.8% identity to an *H. longicornis* tick sequence in the GenBank database (accession no. JQ737090). No *H. longicornis* ticks were positive for *B. miyamotoi*, *B. mayonii*, or *B. microti*.

**Figure:**
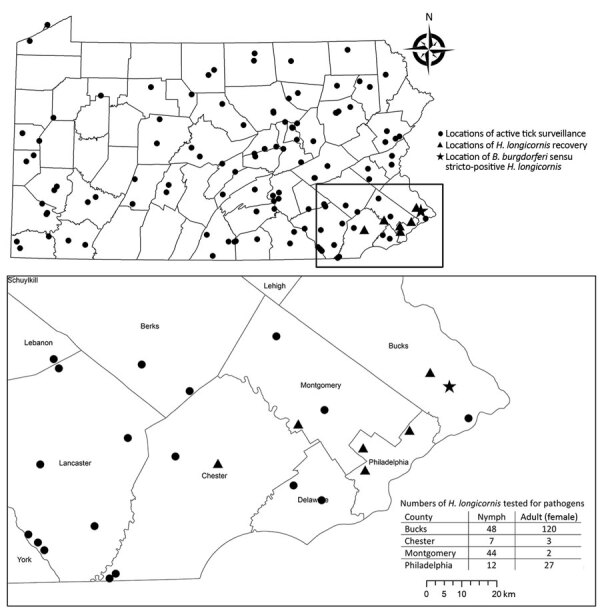
County map of Pennsylvania, USA, and the southeastern region (inset) showing locations of active tick surveillance, where *Haemaphysalis longicornis* ticks were recovered, and where *Borrelia burgdorferi* sensu stricto–positive *H. longicornis* ticks were found, May 1–September 6, 2019. Pennsylvania county map shows 38 counties sampled weekly and an additional 14 counties sampled opportunistically that yielded low tick recovery (*Ixodes scapularis* ticks only).

## Conclusions

We document detection of the Lyme disease spirochete, *B. burgdorferi* sensu stricto, in invasive *H. longicornis* ticks. The overall infection rate of 0.4% was low. In comparison, *B. burgdorferi* sensu lato infection rates in *I. scapularis* ticks collected during the same surveillance period and in the same counties ranged from 16.7% to 57.1% (*M.P.* Price et al., unpub. data). This finding is consistent with recent findings that *H. longicornis* ticks are relatively averse to feeding on white-footed mice (*Peromyscus leucopus*), the primary reservoir of *B. burgdorferi* sensu stricto (*12*). Our findings support laboratory studies demonstrating that *H. longicornis* ticks can acquire *B. burgdorferi* sensu stricto while feeding on experimentally infected mice; however, those studies suggested that *H. longicornis* ticks are unlikely to contribute to transmission of *B. burgdorferi* sensu stricto because infection is lost during molting (*13*). However, refeeding and transmission of Lyme spirochetes by partially-fed ixodid ticks has been documented (*14*).

On the basis of microscopy, we estimated that ≈10% of the host-seeking *H. longicornis* ticks that we recovered were partially fed, suggesting the possibility that transmission could occur before the ticks molt. Of note, however, although we detected *B. burgdorferi* sensu stricto DNA in the tick, we have no evidence to suggest the spirochetes were viable. Unique ecologic traits of *H. longicornis* ticks (e.g., cold hardiness, parthenogenetic reproduction, host generality), which may enable the species’ rapid establishment and high density (*4*), could confound efforts to determine the extent to which the tick may be involved in maintenance of *B. burgdorferi* sensu stricto in nature.

Continued monitoring to identify infested areas is essential, especially in densely populated regions (e.g., southeastern Pennsylvania). Despite limited documentation of *H. longicornis* ticks biting humans in the United States (*15*), findings presented here support continued use of personal protective measures. *H. longicornis* ticks are a vector of human pathogens in its native range; further investigation is needed to determine its potential public health significance in the United States.
